# Valuing biodiversity and ecosystem services: a useful way to manage and conserve marine resources?

**DOI:** 10.1098/rspb.2016.1635

**Published:** 2016-12-14

**Authors:** Rachel D. Cavanagh, Stefanie Broszeit, Graham M. Pilling, Susie M. Grant, Eugene J. Murphy, Melanie C. Austen

**Affiliations:** 1 British Antarctic Survey, High Cross, Madingley Road, Cambridge CB3 0ET, UK; 2 Plymouth Marine Laboratory, Prospect Place, The Hoe, Plymouth PL1 3DH, UK; 3 The Pacific Community (SPC), B.P. D5, 98848 Noumea Cedex, New Caledonia

**Keywords:** ecosystem services, marine biodiversity, ecosystem-based management, conservation, valuation, marine resource management

## Abstract

Valuation of biodiversity and ecosystem services (ES) is widely recognized as a useful, though often controversial, approach to conservation and management. However, its use in the marine environment, hence evidence of its efficacy, lags behind that in terrestrial ecosystems. This largely reflects key challenges to marine conservation and management such as the practical difficulties in studying the ocean, complex governance issues and the historically-rooted separation of biodiversity conservation and resource management. Given these challenges together with the accelerating loss of marine biodiversity (and threats to the ES that this biodiversity supports), we ask whether valuation efforts for marine ecosystems are appropriate and effective. We compare three contrasting systems: the tropical Pacific, Southern Ocean and UK coastal seas. In doing so, we reveal a diversity in valuation approaches with different rates of progress and success. We also find a tendency to focus on specific ES (often the harvested species) rather than biodiversity. In light of our findings, we present a new conceptual view of valuation that should ideally be considered in decision-making. Accounting for the critical relationships between biodiversity and ES, together with an understanding of ecosystem structure and functioning, will enable the wider implications of marine conservation and management decisions to be evaluated. We recommend embedding valuation within existing management structures, rather than treating it as an alternative or additional mechanism. However, we caution that its uptake and efficacy will be compromised without the ability to develop and share best practice across regions.

## 1. Introduction

Approaches to biodiversity conservation based on the notion that nature provides for humans have become increasingly popular in recent years, and notably so since the Millennium Ecosystem Assessment [1]. The value of natural ecosystems to humans is now commonly described using the ecosystem services (ES) concept, where ES are the direct and indirect contributions of ecosystems to human well-being [2,3], and value may be expressed in a range of monetary and non-monetary units, or qualitatively [4]. Despite widespread acceptance and use of valuation and the ES concept, including its uptake by prominent international conservation agreements and bodies, it continues to spark controversy. Much of the ongoing debate is centred on how we place a value on different ES. However, the relationship between ES and the biodiversity underpinning them is also a source of confusion in this field [4].

This paper focuses on the growing interest in valuation of marine biodiversity and ES [5,6]. Humans rely on the ocean for food and biotechnological products, for its vital role in global processes such as nutrient cycling and climate regulation, for its contribution to health and well-being from the leisure and recreation opportunities it provides, as well as for income from activities such as tourism [7,8]. The economic value of coastal and oceanic environments based on tangible outputs such as fisheries production, shipping traffic and carbon absorption was recently calculated at US$2.5 trillion each year with the overall value of the ocean estimated as an asset 10 times this figure [9].

Despite its recognized value to humans, the marine environment is facing increasing anthropogenic pressures from resource exploitation, habitat destruction, pollution and the effects of climate change, with associated widespread declines in biodiversity and threats to key ES [10,11]. Although these threats and declines are widely acknowledged, the ocean presents major challenges for its conservation and management [7,12]. Domestic marine conservation and management measures are essential but marine ecosystems are often trans-boundary, and 60% of the ocean comprises ‘high seas’ and deep seabed beyond national jurisdiction. However, effective international initiatives and regulations are notoriously difficult to implement. Additional complexities are introduced by different geo-political environments, each with their own objectives, organizational structures and frameworks [13].

Historically, efforts focused separately on traditional fisheries management or on biodiversity conservation [14]. Since the 1970s with the emergence of the United Nations Convention on the Law of the Sea (UNCLOS), and international agreements such as the Convention on Biological Diversity (CBD) [15], these pathways have increasingly converged*.* A significant outcome of this shift has been widespread commitment to the ‘ecosystem-based management’ (EBM) approach. This attempts to balance the benefits that people obtain from the ocean against the productivity, health and resilience of its ecosystems [16]. However, full implementation of EBM for marine systems has yet to be achieved [17].

These challenges are exacerbated by substantial practical difficulties in studying the marine environment and associated sampling biases towards certain systems, regions and taxa, which combine to make lack of data and uncertainty key issues [18]. The high levels of connectivity of marine processes, often across vast scales, also brings challenges. For example, fish spawning and nursery grounds are often geographically separated from where adult fishes are later caught by fisheries*.* These issues have ramifications for understanding and addressing the interacting effects of anthropogenic multi-stressors [19], but also for understanding the relationships between biodiversity and ecosystem functioning (BEF). Given the multiple links between BEF and the ES they provide, understanding these relationships is critical for the ES approach and, therefore, for attempts at valuation [4,20–22].

Although interest continues to grow in the use of valuation for the marine environment [5,6,9,23], evidence of its effectiveness is lacking. Given the enormity of the challenges, the urgent need to address them and the scarcity of resources and capacity to dedicate to this, we ask if a valuation approach is appropriate and effective for marine conservation and management.

To begin to address these questions we synthesize and compare the relative merits of adopting a valuation approach in three contrasting ecosystems: the Pacific Island Countries and Territories (PICTs), Southern Ocean and UK coastal seas. Marine ecosystems are of paramount importance to the food security, health and livelihoods of PICT populations [24] and are characterized by a high degree of diversity and endemism. The region supports large offshore industrial tuna fisheries as well as important coastal fisheries. Other threats include climate-induced changes [25,26]. By contrast, the Southern Ocean has no local beneficiaries and supports unique biodiversity in a highly seasonal extreme environment that is experiencing rapid climate change [27]. Although past harvesting was intensive and unregulated, current fisheries are managed by the Commission for the Conservation of Antarctic Living Resources (CCAMLR). UK coastal seas support a high diversity of species of both national and international importance. Fisheries and aquaculture are important sectors in the economy, however, the UK also places value on its other marine ES and has pioneered much of the research in this field [28].

These three regions differ in terms of the benefits they provide to humans, the threats they are experiencing and how they are managed. As such they provide useful contrasting case studies from which to begin to explore the current state of knowledge on the use and efficacy of valuation of biodiversity and ES in marine ecosystems.

## 2. Case studies

Further details on each case study can be found in the electronic supplementary material, table S1.

### (a) Pacific Island Countries and Territories

#### (i) Value of marine ecosystems

The cultures, nutrition and economic development of the PICTs, the 22 island countries and territories within the western and central Pacific region between 25°N and 25°S, are intricately linked to its marine ecosystems. The region has considerable unique biodiversity, in part, owing to its geographical isolation. Coral reefs are integral to PICT livelihoods but there are also large numbers of reefs remote from human pressures; these remain among the best preserved reefs in the world [29]. In addition to providing important habitats, the reefs, sea grass beds and mangroves afford vital coastal protection*.* Although much of the open ocean is relatively unproductive [30], it supports some of the world's largest tuna fisheries [31]. Seamounts are offshore biodiversity hotspots, key for provisioning and other ES [32]. The region's renowned biodiversity attracts tourists, providing important revenue and employment opportunities [33].

The PICTs have among the highest *per capita* consumption of fisheries products in the world. Marine resources represent 50–94% of animal protein in the diet of coastal and urban communities. Much of that comes from inshore fisheries, while offshore tuna fisheries provide 60% of the world's tuna [34]. The fisheries have substantial monetary value (including licensing access of foreign tuna vessels which can provide up to 50% of government revenue) and provide vital employment [35,36].

#### (ii) Main threats

Inshore resources are under pressure owing to increasing population densities [37,38]. Offshore resources face growing fishing pressure through recent increases in vessel numbers and improving technology [34]. Habitat loss from development contributes to reduced natural shoreline protection from mangroves and coral reefs [39]. Coastal erosion is one of the most serious consequences of beach mining, reef blasting and near shore dredging. Other threats include climate change-induced sea-level rise for low-lying atolls [25,26] and increasing tropical cyclone intensity and frequency [40] that may result in population displacement, inshore habitat damage and impacts on national productivity.

#### (iii) Conservation and management

Offshore tuna fisheries fall under the purview of the Western and Central Pacific Fisheries Commission (WCPFC) as well as national processes (see the electronic supplementary material, table S1). The focus is on tuna as the key resource; requirements to conserve biodiversity are a related but separate consideration. Until recently, WCPFC used maximum sustainable yield (MSY) (the theoretical largest amount of catch that can be taken indefinitely from a fish stock) benchmarks to evaluate stock status. However, the importance of tuna for PICTs drove WCPFC proposals to develop more conservative ‘target reference points’ [41]. A key step has been the valuation of target tuna stocks to highlight their direct and indirect monetary value, and the trade-offs involved in management decisions [42,43]. These valuations are combined with consideration of other economic, social and ecosystem objectives. For example, the agreed target reference point for skipjack tuna, which represents around 70% of tuna catches, reflects objectives for income and employment, catch stability and stock sustainability.

Management systems for tuna are also implemented at the sub-regional scale and the national level. These are influenced by international agreements and WCPFC agreements, but also more implicitly the value of ES to PICTs. Traditional management practices, such as permanent or temporary closure of fishing areas, remain strong in the PICTs [26]. Individual PICTs have taken steps to limit by-catch, including implementation of marine protected areas (MPAs) and ‘shark sanctuaries’ with benefits for tourism and biodiversity protection.

#### (iv) Impact and challenges of valuation

Inclusion of the monetary value of provisioning services, combined with consideration of other economic, social and ecosystem objectives, had a direct impact on skipjack tuna with the interim adopted target reference point equating to stock sizes approximately twice that at MSY [43]. While this process did not explicitly account for fishing impacts on biodiversity, it has implicit benefits for the wider ecosystem, while recognizing, for example, aspects of cultural services. As an example for the inshore region, estimates of loss in economic value arising from coastal erosion because of aggregate mining on Majuro Atoll were found to outweigh the contribution of mining to the economy. However, while the importance of other coastal ecosystem values was recognized they were not estimated in this study [44].

#### (v) Future research

More research is required in terms of understanding BEF relationships, how these may be affected by change, the implications for ES and linking these more explicitly to management decisions. While valuation of provisioning services has begun, that for other ES is yet to be explicitly included. Other ES such as climate regulation and nutrient cycling are important. Increased protection from climatic events is a priority, combined with the importance of adapting to climate-induced sea-level rise. For example, maintaining and enhancing coral reef structure and function may be a practical and cost effective solution to hazard mitigation and adaptation [45].

In terms of the MPAs, wider benefits for regional tuna stocks (as opposed to less mobile reef fishes) may be limited by their highly migratory nature [46], particularly where fishing effort merely redistributes rather than reduces. For nations reliant on foreign vessel licence fees, as many PICTs are, denying access through large-scale MPAs has significant implications. There is, therefore, a need to explore the balance of different ES within decision-making.

#### (vi) Summary

This is a region where the economic value of the fishery resources is high and consideration of other ES is just beginning to be explored. While a range of methods have been employed, from market-based valuations through to survey-based and stated preference techniques [47,48], a more comprehensive valuation is required to capture the PICT context, enable development, ensure food security and allow the conservation of biodiversity [49,50].

### (b) Southern Ocean

#### (i) Value of marine ecosystems

The Southern Ocean, defined here as the area encompassing the Antarctic Circumpolar Current (ACC) and regions to the south, influences global climate and biogeochemical cycles and supports internationally important fisheries [51]. Its global importance is recognized through the Antarctic Treaty System (ATS), which provides a high level of protection and management through international agreements. Grant *et al*. [27] identified key ES including provisioning services such as fishery products and the growing high-end market for krill oil as a health supplement; regulating services such as climate regulation (e.g. sequestration of CO_2_ and regulation of global sea level); supporting services such as nutrient cycling and primary productivity; and cultural services such as tourism and iconic wildlife (e.g. penguins, whales, seals and albatrosses). The role of biodiversity in ecosystem functioning in the polar regions is explored elsewhere in this volume [22]. The Southern Ocean does not border a permanently inhabited landmass. This lack of local beneficiaries means the provisioning services have markets predominantly in East Asia, North America and Europe, whereas the regulatory and supporting services benefit human populations at the global scale [27].

#### (ii) Main threats

Southern Ocean ecosystems were subject to two centuries of largely unregulated harvesting (e.g. Antarctic fur seals, baleen whales and finfish [52]). Sixteen nations currently operate here, including fisheries for toothfish, mackerel icefish and Antarctic krill. The krill fishery currently operates at a low level with a catch limit equivalent to only 1% of the estimated biomass (60.3 × 10^6^ t) and actual catches lower than this [53]. However, its potential to become one of the largest fisheries in the world has been highlighted [54]. Toothfish and mackerel icefish are likely to be fully exploited and toothfish depleted in some areas of the Indian Ocean through illegal, unregulated or unreported (IUU) fishing [55].

The region is currently undergoing unprecedented climate-driven changes with local as well as far-reaching consequences [56]. The physical dynamics of Southern Ocean water masses are rapidly changing owing to atmospheric changes including the loss of stratospheric ozone, and are, in turn, affecting the physical and biological carbon pumps; ocean temperatures are increasing; sea ice duration and extent is changing; and ocean acidification is especially pronounced in polar waters [57].

#### (iii) Conservation and management

Governance of the Antarctic is unique and comprises a set of international agreements within the ATS. The Protocol on Environmental Protection to the Antarctic Treaty regulates all human activities except for fishing, and recognizes the intrinsic value of Antarctica beyond the financial value of its exploitable resources [58]. Within the ATS, fishing activities are managed by CCAMLR, which has been described as a pioneer of the ecosystem approach to fisheries management [13]. The CCAMLR Convention was the first international fisheries management agreement to set out specific ‘principles of conservation’ relating to the wider ecosystem, as an integral part of its harvesting regime (CCAMLR Convention, Article II) [59]. These principles are precautionary and reflect EBM goals in requiring managers to maintain the productivity of harvested populations, to maintain ecological relationships (between the harvested species and any dependent or related species) and to prevent irreversible change (see the electronic supplementary material, table S1). Management decisions must, therefore, consider the trade-offs between current and future catches, and the more general benefits of a healthy ecosystem [27]. However, in the context of CCAMLR's decision-making, fisheries management and related conservation principles are currently being considered separately from information and decisions related to other Southern Ocean ES.

#### (iv) Impact and challenges of valuation

Despite ongoing monitoring and data gathering efforts by national science programmes, and coordinated multi-national programmes, such as the CCAMLR Ecosystem Monitoring Programme (CEMP), the Census of Antarctic Marine Life (CAML) and the Scientific Committee on Antarctic Research (SCAR) Biogeographic Atlas of the Southern Ocean [60], the Southern Ocean has not been the subject of a detailed or formal regional ecosystem assessment, information on ES is lacking and valuation tends to be based on the economic value of Southern Ocean fisheries [27].

#### (v) Future research

The economic value of the fisheries should be considered alongside the value of other ES that the target species provide. For example, in the case of krill this includes their role as a key species in the food web, including contribution to predator production [22], and other intrinsic (i.e. non-monetary) values [27].

Although CCAMLR has resolved to increase consideration of climate change impacts, guidance on how this can be achieved in practice is still being developed. Furthermore, the region is under-represented in global ecosystem assessments such as the MEA [61] and the United Nations Environment Programme's (UNEP) Regional Seas synthesis and global environmental outlook [62,63]. Further understanding of the wider benefits obtained from Southern Ocean ES and biodiversity would help ensure that their value is adequately recognized in decision-making at regional and global scales [58], and would also help improve understanding of the consequences of change in these ecosystems [22].

#### (vi) Summary

CCAMLR's management approach incorporates a range of ecosystem-based trade-offs, however, there is currently a lack of information on the value of ES in this region. Concepts of relative and intrinsic value exist within the ATS, and could be further used in informing decision-making. In particular, the valuation of biodiversity and ES could help to articulate specific management objectives within CCAMLR and to communicate these effectively to other regional and global organizations. This could also facilitate the consideration of all ES (including those relating to, e.g. climate regulation, ocean circulation, tourism), and the biodiversity underpinning them, as part of CCAMLR's EBM framework.

### (c) UK coastal seas

#### (i) Value of marine ecosystems

UK coastal seas support a high biodiversity that underpins a range of ES and benefits of significant value to UK society and internationally. These include food supplies and contributions to climate regulation (e.g. through high carbon sequestration into salt marshes and seagrass beds, and transport of pelagic carbon offshore into deeper layers), and human health and well-being (e.g. by providing space for recreational activities) [28,64]. Fisheries and rapidly increasing aquaculture are important sectors in the economy [65,66]. However, the UK also relies on its marine biodiversity for other biotic raw materials, such as seaweeds for energy and food additives; regulating services such as bioremediation of waste and disturbance prevention; and cultural services such as education and research.

Beaumont *et al*. [64] carried out an ES approach to determine the economic value of UK marine biodiversity. They concluded that biodiversity loss is likely to cause unpredictable changes in the provision of ES because of lack of knowledge of the multiple links between biodiversity, functions and the various ES they provide. For example, vulnerable biogenic habitats provide nursery and refuge areas for other species that in turn are important for other ES. In 2011, a comprehensive UK-wide National Ecosystem Assessment (NEA) was undertaken which included valuation (monetary and non-monetary) of the ecosystems and services they provide. A key finding was that the UK is comparatively data rich with regard to BEF relationships [28,67], for example, food webs and biogeochemical cycling.

#### (ii) Main threats

The NEA highlights a number of threats to UK coastal biodiversity. These include unsustainable fishing practices which can affect food provision and also impact other ES by damaging habitats and through unwanted by-catch. Pollution from land and freshwater run-off was also highlighted as an issue, particularly through emergent new chemicals such as pharmaceuticals and microplastics, and pollution from shipping accidents [28]. Global climate change is already having an effect on UK marine biodiversity with expected impacts on most ES.

#### (iii) Conservation and management

The UK marine environment is governed by a complex web of national, European Union (EU) and international policy and legislation that increasingly aim towards sustainable management of the ocean and its biodiversity (electronic supplementary material, table S1) [68]. Fisheries management is progressing towards MSY under the EU Common Fisheries Policy (CFP). The EU Marine Strategy Framework Directive (MSFD) aims at achieving Good Environmental Status (GES) in EU marine waters through regular, national assessments of key descriptors of the marine environment (e.g. biodiversity and commercial fishes but also marine litter and underwater noise) and implementation of necessary management measures. Measures undertaken in the UK include designation of MPAs and implementation of planning and licencing for all human activities in the marine environment. While many of the policies mention ES, they do not specify marine management in an ES framework, although the UK has used an ES approach within these, for example, to analyse the costs of not reaching GES [69]. Although the UK aims to take an ecosystem approach in developing national marine plans [70], the focus is on sustainable use of marine space within each Marine Plan area (electronic supplementary material, table S1) rather than management and planning for ES.

Indicators to allow changes in ES to be quantified were developed for a case study (Dogger Bank) on using the ES approach in UK marine management. This study demonstrates the use of indicators, the value of marine ecosystems to the public and the need to consider wider stakeholder views [71,72].

#### (iv) Impact and challenges of valuation

Valuation approaches in the UK have successfully led to changes in policy. In 2006, the first monetary valuation of UK marine biodiversity provided evidence that supported development of marine legislation (electronic supplementary material, table S1); and the results of the NEA are evident in the UK Post-2010 Biodiversity Framework and its ongoing implementation. Valuation approaches are rapidly permeating into different areas of marine and coastal management, particularly as the UK embraces natural capital accounting for ES. The challenge now is to provide operational tools to support ES assessments and valuations, and resources to collect the required data.

Difficulties in determining non-use values for marine ES can often be to problems faced by the public in assessing their value. Although this is particularly true for marine systems, Jobstvogt *et al*. [73] demonstrated the public's willingness to pay to protect deep sea habitats in Scotland and showed that the value of such habitats can be assessed using discrete choice questionnaires. However there are still only a small number of such studies for marine habitats that can be used to support valuation of ES.

#### (v) Future research

The examples above demonstrate major advances in using valuation in UK marine management. Yet possibly owing to a lack of legislation focused on managing multiple and interacting ES, there is no driver to ensure collection of new data, or organization of existing data, to facilitate such assessments. A key finding of the NEA was that the links between biodiversity, processes and services in UK waters need to be better understood and quantified [28,67]. While this is of global truth it is highlighted here to illustrate that although large datasets are available they are not always accessible, exhaustive, nor spatially matched in order to deliver quantitative, spatial data on ES.

#### (vi) Summary

The UK has developed methodological approaches and expertise to assess marine ES and value their benefits. This is combined with a strong interest and willingness of policy makers to use these assessments to support complex decisions on the sustainable use of the marine environment. Continuing efforts to ensure integration of these approaches into the conservation and management of resources throughout UK coastal waters are required.

## 4. Discussion

This study, unique in its review of valuation across different marine systems, has revealed a diversity in approaches with varying rates of progress and success. The contrasting case studies together highlight benefits and challenges in using valuation approaches in these different regions. Consideration of the monetary value of fisheries in the PICT has successfully demonstrated that this helps to focus management decisions. In the Southern Ocean, resource management encompasses a broader range of ecosystem considerations, but does not currently include any specific valuation. Work in the UK indicates the steps needed to further these approaches so that wider trade-offs, including the implications of biodiversity changes resulting from both human impacts and natural variability, can be considered explicitly and more routinely in policy and management.

There is a tendency for valuation in marine systems to focus on specific ES (often the harvested species) rather than biodiversity. The PICT case study demonstrates advantages to this relatively simple valuation when brought into an ES context. Here this has led to a more conservative target reference level for tuna and catalysed a process of considering wider ES. However, focusing on the economic value of target species can often result in a failure to consider other ES that the species may provide, and of the underpinning biodiversity and ecosystem structure and functioning. All of these aspects are probably contributing to supporting the fishery as well as providing wider ecosystem benefits.

Many ES do not easily lend themselves to the assignment of a monetary value and in some cases a monetary value may not be appropriate [4]. Direct monetary valuation may even potentially put species at risk, particularly if the species is rare. Increased value can stem from rarity, or valuable species can become rare because of increased consumer demand [74]. That said, recognized high value can also promote conservation efforts for rare species and habitats that are iconic and valued for leisure and recreation (such as cetaceans, seabirds and coral reefs). Without some understanding of ‘value’, less obvious ES such as coastal defence and bioremediation of waste may be ‘lost’ in management decisions or afforded less attention than they merit. Other difficulties include the concept of attaching values to non-use benefits, for example, from supporting or cultural ES. Despite such challenges the UK case study showcases comprehensive work that incorporates both monetary and non-monetary values, and that is increasingly permeating policy and management.

The case studies show that valuation approaches can be effective at national or small-scale regional levels, for example, in enabling different uses of an Exclusive Economic Zone to be considered, and in helping with the implementation of tools such as marine spatial planning, as highlighted in the UK case study. In the Southern Ocean (almost entirely high seas except for sub-Antarctic islands under national jurisdiction), fisheries management and related conservation principles are currently being considered in isolation from information related to other ES such as climate regulation. Here, as has begun in the PICT (e.g. where highly migratory fish stocks and issues of food security are involved), a valuation approach could be useful in incorporating wider ES into decision-making.

**Figure 1.**
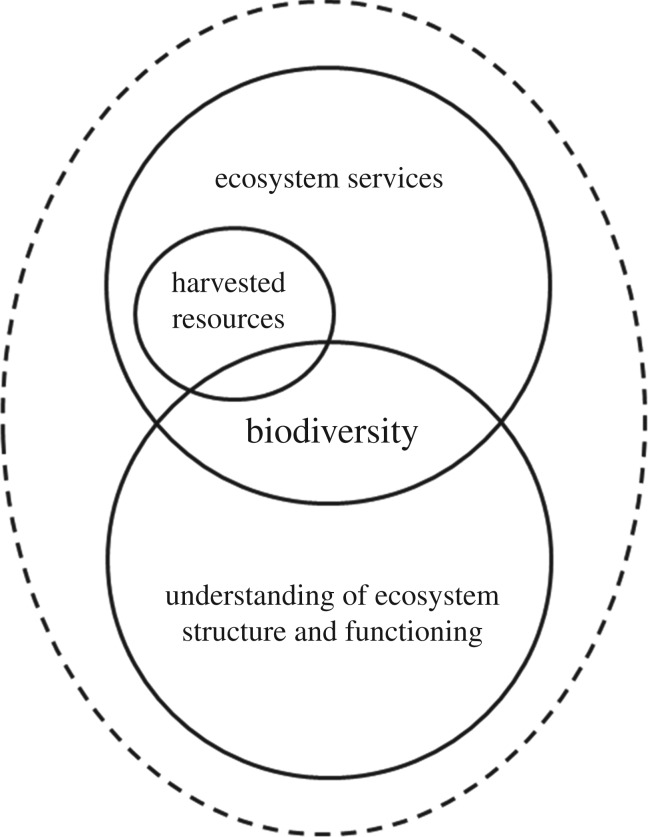
Conceptual diagram showing the interacting components (solid circles) that should be considered as part of the valuation of marine ecosystem services (ES) and biodiversity. Biodiversity is central to all of these components, underpinning the delivery of all services provided by the ecosystem, including specific provisioning services such as the harvesting of marine living resources. An understanding of the structure and functioning of ecosystems requires information on biodiversity [4,22], and is crucial to valuation. The case studies presented here demonstrate that these different components have been considered to varying extents in different regions. This may be because specific priorities or management objectives are being addressed, or may simply reflect varying rates of progress. Broadly speaking, the PICTs would currently be positioned mostly in the harvested resources component; the Southern Ocean centred on harvested resources but overlapping with aspects of ecosystem structure and functioning; and the UK would encompass all components, but linking less well to management of harvested resources for example. Valuation approaches for individual components remain useful, however, it is helpful to consider all of these components as part of a broader, overarching concept (dashed line), reflecting the critical relationships and interactions between biodiversity, ES and an understanding of ecosystem structure and functioning.

However, the need to better understand and incorporate existing knowledge on BEF relationships into the ES approach is fundamental to the success and rate of progress we describe in this study (electronic supplementary material, table S1). Furthermore, consideration of these relationships is not linking as effectively as it could with conservation and management in any of the case study regions. In light of these findings, we propose a conceptual view to valuation that is centred on the critical interactions between biodiversity, ES and an understanding of ecosystem structure and functioning (figure 1). The central role of biodiversity in underpinning these interactions and the delivery of all ES is pivotal to this concept.

This conceptual view could provide a useful means to highlight knowledge gaps and key uncertainties, and to define priorities for addressing these for any given region. The role of modelling is likely to be of paramount importance to support this conceptual approach, and the input of better scientific understanding to all its components. For example, development of existing coupled hydrological, ecological and fisheries models, linking them to economic models and decision support tools (e.g. multi-criteria analysis and probabilistic graphical models) and then making them regionally transferable would provide much needed tools [75]. Better collation and use of existing data, plus guided monitoring focusing on ES indicators could also improve the developed decision support tools.

The case studies also highlight challenges such as the need to consider trade-offs in decision-making (e.g. MPAs in the PICT); the need to more clearly define objectives within EBM (e.g. in CCAMLR); and the challenges of interdisciplinary collaboration (including ecologists, modellers, economists, social scientists and policy makers) (e.g. much of the UK work). There is a need to describe and quantify the different elements for consideration in terms that can be understood across multiple sectors, stakeholders and decision-makers. We suggest that valuation conducted within this overarching concept (figure 1) could bring this commonality. Providing the means to improve understanding of the wider implications of decisions in this way will help to bridge the divide between resource management and biodiversity conservation.

Uptake by stakeholders and decision-makers is ultimately required to make this operational. Therefore, rather than treating the valuation of ES and biodiversity as an alternative or additional mechanism, we recommend a multidisciplinary, flexible approach that embeds it in existing resource management frameworks. This would ensure that aspects such as culture, history, data availability, capability and context are accounted for. Further case studies and broad-scale comparative analyses are necessary to provide proof of concept from different regions. Given the general scarcity of resources and capacity, sharing ‘lessons-learned’ across regions would be beneficial in identifying best practice (e.g. approaches that may be particularly relevant for high seas areas), and in helping to tailor approaches to the particular ecological, social, economic and cultural context.

We conclude that a broad approach to valuation is required such that the foundational role of biodiversity in sustaining the value of ecosystems [4] can be brought explicitly into decision-making. By considering the critical relationships between biodiversity, ES and an understanding of ecosystem structure and functioning, this approach provides a more comprehensive recognition of value and as such has strong potential to contribute effectively to the conservation and management of marine resources.

## Supplementary Material

Table 1. Marine biodiversity and ecosystem services: value, threats and conservation in the three case study regions: Pacific Island Countries and Territories, Southern Ocean and UK coast
