# Supplementary material for: Valuing biodiversity and ecosystem services: a useful way to manage and conserve marine resources?
Source: Proc Biol Sci. 2016 Dec 14;283(1844):20161635. doi: 10.1098/rspb.2016.1635 (PMC5204147; doi:10.1098/rspb.2016.1635)
Supplement: Table 1. Marine biodiversity and ecosystem services: value, threats and conservation in the three case study regions: Pacific Island Countries and Territories, Southern Ocean and UK coast [file rspb20161635supp1.docx]

**Table 1. Marine biodiversity and ecosystem services: value, threats and conservation in the three case study regions: Pacific Island Countries and Territories, Southern Ocean and UK coast.**

The three regions differ in terms of the benefits they provide to humans, the threats they are experiencing and how they are managed. They provide useful contrasting case studies from which to begin to explore the current state of knowledge about using a valuation approach (methods, challenges, impacts) in marine conservation and management. This table contains additional detail that can be cross-referenced with each case study and the comprehensive set of regional reference material therein. Note that additional references (i.e. those not cited in the case studies: 76-85) have been added to this table where appropriate.

|  | **Pacific Island Countries & Territories** | **Southern Ocean** | **UK coastal seas** |
| --- | --- | --- | --- |
| **Value**  **of marine biodiversity and ecosystem services** | *Provisioning services:*  Locally/regionally: marine resources = 50-94% of animal protein in PICT diet.    Main regional fisheries: coastal. ~>USD$360 million value for subsistence and commercial fisheries. Coastal catches ~150,000t. Local employment opportunities.  Offshore commercial fisheries: 60% of world’s tuna. Tuna catches (2014) ~2.8 million mt, value ~ USD$5.8 billion. Four key species (skipjack, yellowfin, bigeye, albacore). Significant income from licensing of foreign vessels within PICT EEZs (up to 50% of government revenue in some countries). Vital employment, incl. processing canneries, fishing vessel crew, fishery observers.  Together, coastal and offshore fisheries represent ~3.7% of global marine capture fisheries by weight (78).  Aquaculture: struggled for economic sustainability. Some successes (e.g. in PNG) aiding food security and providing livelihood options.  *Cultural services:*  Growing sectors such as ocean-based tourism provide ~USD 2.3 billion, or 7% of regional GDP, to the economies of PICTs as well as offering valuable employment opportunities (76). Spiritual appreciation and connection with marine ecosystems, including sacred sites, and basis for significant cultural and maritime heritage.  *Regulating and supporting services:*  Climate regulation, e.g. mangroves fix and store significant amounts of carbon and play an important role in carbon sequestration. They also absorb pollutants.  Primary production and nutrient cycling important both globally and regionally, e.g. in maintaining food webs that support offshore tuna fisheries.  Habitat provision and coastal protection, e.g. coral reefs, seagrass beds and mangroves provide fish nursery habitats, as well as providing protection from storm surges and waves, and prevention of coastal erosion.  *Biodiversity underpinning ES*:  In addition to cultural and aesthetic benefits, the relationship between BEF in the PICTs is crucial to understanding coastal and offshore production. Seamounts are biodiversity hotspots and a key habitat for provisioning services and other ES. Nearshore biodiversity plays a role in resilience to climate change. | *Provisioning services:*  Antarctic krill (*Euphausia superba*):  Current annual catches of Antarctic krill valued at ~USD$241 million. 2014/15 catch of 225,645t (83) (equivalent to 0.27% of global marine capture fisheries by weight in 2014/15 (78)). Products sold mainly in US, Asian & European markets (for human (including health supplements from krill oil) and animal consumption).  Patagonian and Antarctic toothfish (*Dissostichus* spp.):2014/15 catch of 15,892t (83). High value fish products sold mainly in Japan & US.  Additional economic importance for governments which  generate revenue from fishing licences, and for port states, and others involved in processing or related industries.  *Cultural services:*  Tourism - a small minority of the global population (~35,000 per year). Cultural and aesthetic benefits include iconic species e.g. penguins, whales and seals. Scientific research is internationally important ~ 6,000 scientific/support personnel on the continent.  *Regulating and supporting services:*  Major role in climate regulation, e.g. global ocean circulation system, major sink of atmospheric CO_2,_ regulation of global sea level.  Total primary production in this region is equivalent to ~3.5% of total world ocean productivity. Highly variable with regions of high productivity including the Polar Front and highly seasonal Marginal Ice Zone.  Important role in global nutrient cycling and regional role in maintaining Southern Ocean food webs, including harvested species.  *Biodiversity underpinning ES*:  In addition to its cultural and aesthetic benefits (e.g. iconic species), the role of biodiversity in ecosystem functioning in the polar regions is key to understanding the value of its ES. Aspects of ecosystem structure and function are considered by CCAMLR and explored in more detail elsewhere in this volume. | *Provisioning services:*  Fisheries and aquaculture are important sectors in the UK economy with landings valued at £861 million (in 2014) and £590 million (aquaculture in 2012) respectively.  Fisheries: 754,992t in 2014, 0.9% global marine capture fisheries by weight (78). Value £861 million. Demersal fisheries are particularly valuable in UK landings, e.g. cod, haddock, plaice, whiting, pollack and soles. 11,845 fishermen employed.  Aquaculture production: 167,300t of finfish and 238,00t of molluscs in 2014 which is 2.6 and 0.1% respectively of global farmed species. 3,231 employees.  The UK also relies on its marine environment for other biotic raw materials, such as seaweeds for energy, fertiliser and food additives.  *Cultural services:*  Tourism, recreation such as scuba diving or snorkelling, well-being, cultural heritage and identity, education, research.  *Regulating and supporting services:*  Climate regulation, e.g. high carbon sequestration by coastal algae, salt marshes and seagrass beds; transport of pelagic carbon offshore into deeper layers.  Bioremediation of waste; disturbance prevention and alleviation through saltmarshes, coastal mudflats and sandbanks.  Nutrient cycling is important due to significant nutrient input from rivers, groundwater and offshore waters.  Habitat provision by organisms, e.g. kelp forests provide nursery habitats for juvenile fish which supports provisioning services.  *Biodiversity underpinning ES*:  The UK is comparatively data rich with regards to both marine biodiversity and ecosystem functioning relationships, e.g. food webs and biogeochemical cycling. However biodiversity loss is likely to cause unpredictable changes in the provision of ES due to lack of knowledge of the multiple links between biodiversity, functions and the ES they provide. |
| **Main threats** | Climate change and extreme weather events e.g. sea level rise; increase in cyclone intensity/frequency. Cyclones, coral bleaching, mangrove removal for development and other impacts (incl. beach mining, reef blasting), have reduced natural shoreline protection, increasing exposure to these events and causing coastal erosion.  Increasing pressure on coastal resources has led to calls to refocus effort offshore (77). However, while generally exploited sustainably (bigeye stock assessed as overfished but otherwise main tuna stocks within desired limits), offshore resources face growing pressure through increasing numbers of vessels/improving technology.  Fishing practices may cause habitat damage. E.g. increased use of drifting fish aggregation devices (FADs) - may wash up and damage reefs.  IUU fishing is considered low due to national/regional efforts. However, the loss in rent for Forum Fisheries Agency (FFA) member PICTs is ~ USD$153 million (82).  Nearshore pollution from domestic and farming waste. | Currently undergoing unprecedented climate- driven changes with local as well as far-reaching consequences. Physical dynamics of water masses are rapidly changing due to atmospheric changes incl. loss of stratospheric ozone, and are affecting physical and biological carbon pumps; increasing ocean temperatures; changing sea ice extent; and ocean acidification is especially pronounced in polar waters.  Increasing catch levels and potential overexploitation of fish stocks (although most catch limits are highly precautionary, and total catches remain low).  Habitat loss/destruction through interaction with long-line fishing gear.  By-catch of non-target species, and incidental mortality of marine mammals and seabirds.  IUU fishing, e.g. for high-value toothfish.  Pollution from local point sources, e.g. fuel spills, chemical contamination and sewage as well as long-range pollutants originating outside Antarctica (84). | Effects of climate change already impacting UK marine biodiversity with expected impacts on ES. E.g. fish community structure has been altered through changes in distribution, migration, recruitment, growth and phenology of food source.  Nutrient cycling may be altered due to climate change e.g. when changes in water temperature lead to new current regimes as well as water stratification.  Unsustainable fishing can reduce food provision but also impacts other ES by damaging habitats and through unwanted bycatch. E.g. demersal fisheries declined from 1948 to 2000.  Pollution, from land and freshwater run off, and particularly through emergent new chemicals such as pharmaceuticals and microplastics, and pollution from shipping accidents. |
| **Conservation and management** | WCPFC and PICTs tuna fishery management aims for sustainable resource use. Until recently, WCPFC used MSY benchmarks to evaluate stock status, now has begun adopting more conservative “target reference points”.  The WCPFC also manages key vulnerable species. This includes separate regulations on mitigation of non-target bycatch e.g. seabirds, turtles, sharks.  Management systems are also implemented at the sub-regional scale (e.g. the FFA, and the Parties to the Nauru Agreement (PNA) within whose waters most purse-seine fishing takes place) and the national level. These are influenced by international agreements, but also more implicitly the value of ES to PICTs.  Individual PICTs have taken steps to limit bycatch impacts, including implementation of marine protected areas (MPAs) and “shark sanctuaries” within their EEZs. Palau, for example, has significant income from the tourist trade and their shark sanctuary, which bans commercial shark fishing, attracts dive tourists.  Traditional management practices, such as permanent or temporary closure of fishing areas or bans on catching some species, remain particularly strong in the Pacific. | Governed by the ATS - legally binding in signatory nations. Within this, mineral exploitation is prohibited and specific rules exist for scientific and tourist operations. Fishing is managed by CCAMLR’s ecosystem approach with precautionary conservation principles. These include balancing conservation with rational use; protecting dependent/related species and restoring depleted populations/stocks; avoiding changes that are potentially irreversible.  Other relevant conventions: International Convention for the regulation of Whaling; Convention for the Conservation of Antarctic Seals (85).  CCAMLR’s conservation measures include precautionary catch limits (krill and toothfish), gear restrictions, and open/closed areas and seasons. Measures to minimise potential impacts on krill predators include dividing krill catch limit across subareas, and development of a feedback management system. CCAMLR has established one MPA (south of the South Orkney Islands). Additional MPAs are under consideration. Marine reserves have also been established at sub-Antarctic islands under national jurisdiction.  Bottom trawling and gill-netting are prohibited throughout the CCAMLR Convention Area. Tackling bycatch, particularly seabird mortality, is part of the regulatory framework for long-line fisheries.  Fishing vessels have to carry observers for some/all of their operations. Measures to tackle IUU incl collaboration with other RFMOs, satellite-linked vessel monitoring systems, and a catch documentation scheme. | Multiple national, EU and international policy and legislation. Historically instruments have been sectoral, either concerned with resource extraction e.g. EU Common Fisheries Policy (CFP) for fisheries management or with conservation (79) e.g. EU Habitats and Birds Directives (80, 81).  The CFP, EU Marine Strategy Framework Directive (MSFD) and EU Biodiversity Strategy take a more cross-sectoral view. E.g. MSFD is focused on the natural environment and biodiversity whilst allowing sustainable use of ES (79).  UK legislation includes the Marine and Coastal Access Act (MCAA) to sustainably manage and plan use of the marine environment. For some fish stocks, such as cod and sole, special recovery zones have been implemented (MMO).  The Marine Policy Statement (2011) is the framework for the preparation of holistic marine plans for all UK seas taking into consideration all uses and stakeholders.  Many of these policies mention ES, but do not specify marine management in an ES framework. E.g. use of space for fisheries is acknowledged in the Marine Plans (note that designated regional areas are known as Marine Plan Areas), but fisheries management is not explicitly undertaken within such a holistic framework, but through separate sectoral elements of MCAA to comply with the CFP. |
| **Methods of valuation used to date** | Direct economic value of the fishery resources.  Market-based valuation through survey-based and stated preference techniques.  Specific studies e.g. economic value of coastal ecosystem to assess impacts of beach mining. | Direct economic value of the fishery resources. | Economic valuation of the goods and services provided by UK marine biodiversity by combining both ecological and economic approaches (using market data and contingent valuation).  National Ecosystem Assessment (NEA): data on ES collated for all UK habitats including marine.  Specific case studies: e.g. Dogger Bank: use of indicators to allow changes in ES to be quantified and socio-economic survey to assess how the public valued this ecosystem. |
| **Challenges to valuation** | Lack of data on many of the ES and the BEF underpinning them. The spatial scale and diversity of the issue (incl. many different islands, many different issues, many different stakeholders, multiple groups working in the different disciplines). | No detailed or formal ecosystem assessment exists.  Lack of data on many of the ES and the BEF underpinning them, e.g. regulating and supporting services.  Remote beneficiaries – value to whom? | Lack of data for specific ES as well as methods to assess them for example biological control – checks and balances/ resilience. |
| **Impact of existing valuation** | Influenced selection of tuna target reference points, implying more conservative fishing levels than ‘default’ MSY – for skipjack tuna = twice MSY. While this process did not explicitly take into account impacts of fishing on biodiversity the target level has implicit benefits for biodiversity and the ecosystem, while recognising, for example, aspects of ES such as cultural services.  Estimates of the loss in economic value arising from aggregate mining on Majuro Atoll (ranged from USD$88-373 million) were used to demonstrate that the value of the coastal ecosystem outweighs the contribution of mining to the economy. | Although trade-offs between the benefits obtained from harvesting and the potential impacts on the wider ecosystem are a major  component of CCAMLR’s decision-making process, particularly with respect to the krill fishery, these are not currently undertaken specifically as part of an ES/valuation approach. | Valuation approaches used in the UK have successfully led to changes in policy:  The first monetary valuation of UK marine biodiversity provided evidence that supported development of integrated marine legislation within the Marine and Coastal Access Act (2009);  The ES approaches of the NEA are evident in the UK Post-2010 Biodiversity Framework, and its ongoing implementation;  The value of the ES approach is rapidly permeating into different areas of marine and coastal management, particularly as the UK embraces natural capital accounting for ES. |
| **Future research** | Broaden valuation beyond provisioning services to address regional priorities incl protection from climatic events/adapting to climate-induced change. E.g. maintaining/enhancing coral reef structure and function may be an effective solution to hazard mitigation and adaptation.  More research on BEF relationships, how these may be affected by change, implications for ES, and linking these to management decisions. E.g. how pelagic food web structure and function may respond to climate change, how this may impact the tuna fishery and wider ecosystem.  Explore use of ES/valuation to:  Help with trade-offs in decision-making e.g. MPAs vs licence fees; coastal development vs protection;  Better articulate value of PICTs in an international context. | A comprehensive ecosystem assessment, including consideration of ES and their benefits.  Improve understanding of BEF relationships, how these may be affected by change, and their relationship with ES. E.g. changes to the krill-based food web, how this may impact the fishery and wider ecosystem.  Explore use of ES/valuation with CCAMLR to:  Improve knowledge about connections between ES and CCAMLR objectives e.g. the role of supporting services in the maintenance of harvested, associated and dependent populations.  Consider wider ES currently not represented in fisheries-related decision-making e.g. associated / dependent biodiversity; ocean circulation.  Ensure the value of the Southern Ocean is adequately recognised globally, and to help CCAMLR coordinate with other organisations regionally and globally. | The links between biodiversity, functions and services in UK waters need to be better understood and quantified, e.g. to improve understanding of the effects of biodiversity loss on ES.  Efforts are needed towards legislation focused on managing multiple and interacting ES to help ensure collection of new data and organisation of existing data (large datasets are available but are not always able to deliver required quantitative, spatial data on ES).  Exploration of the use of ES/valuation combined with EBM for regional management of UK coastal seas within the Marine Plans. |
